# The effects of low and high glycemic index foods on exercise performance and beta-endorphin responses

**DOI:** 10.1186/1550-2783-8-15

**Published:** 2011-10-20

**Authors:** Athanasios Z Jamurtas, Trifon Tofas, Ioannis Fatouros, Michalis G Nikolaidis, Vassilis Paschalis, Christina Yfanti, Stefanos Raptis, Yiannis Koutedakis

**Affiliations:** 1 University of Thessaly, Department of Physical Education and Sport Science, Karies, 42100, Trikala, Greece; 2 Institute of Human Performance and Rehabilitation, Centre for Research and Technology - Thessaly, Greece; 3 University of Thrace, Department of Physical Education and Sport Science, 69100, Komotini Greece; 4 Semmelweis University, Budapest, Hungary; 5 University of Wolverhampton, School of Sports, Performing Arts and Leisure, Gorway Road, Walsall, WS1 3BD, UK

**Keywords:** Glucose, insulin, opioids, training, food

## Abstract

Τhe aim of this study was to examine the effects of the consumption of foods of various glycemic index values on performance, β-endorphin levels and substrate (fat and carbohydrate) utilization during prolonged exercise. Eight untrained healthy males underwent, in a randomized counterbalanced design, three experimental conditions under which they received carbohydrates (1.5 gr. kg^-1 ^of body weight) of low glycemic index (LGI), high glycemic index (HGI) or placebo. Food was administered 30 min prior to exercise. Subjects cycled for 60 min at an intensity corresponding to 65% of VO_2_max, which was increased to 90% of VO_2max_, then they cycled until exhaustion and the time to exhaustion was recorded. Blood was collected prior to food consumption, 15 min prior to exercise, 0, 20, 40, and 60 min into exercise as well as at exhaustion. Blood was analyzed for β-endorphin, glucose, insulin, and lactate. The mean time to exhaustion did not differ between the three conditions (LGI = 3.2 ± 0.9 min; HGI = 2.9 ± 0.9 min; placebo = 2.7 ± 0.7 min). There was a significant interaction in glucose and insulin response (*P *< 0.05) with HGI exhibiting higher values before exercise. β-endorphin increased significantly (*P *< 0.05) at the end of exercise without, however, a significant interaction between the three conditions. Rate of perceived exertion, heart rate, ventilation, lactate, respiratory quotient and substrate oxidation rate did not differ between the three conditions. The present study indicates that ingestion of foods of different glycemic index 30 min prior to one hour cycling exercise does not result in significant changes in exercise performance, β-endorphin levels as well as carbohydrate and fat oxidation during exercise.

## Background

Carbohydrate ingestion prior to exercise has been shown to affect metabolic responses and performance [1]. It is suggested that carbohydrate feeding prior to exercise provides additional supplies for oxidation, results in increased muscle glucose uptake and reduced liver glucose output during exercise [2] and the enhanced blood glucose availability may preserve muscle glycogen stores [3].

β-endorphin is one of the peptides that has been suggested to play a role in glucose metabolism at rest [4,5] and during exercise [6-9]. β-endorphin is an opioid peptide representing the C-terminal 31 amino acid residue fragment of pro-opiomelanocortin. Data indicates that stress is a potent inducer of β-endorphin release and it is well known that exercise of sufficient intensity and duration elevates its circulating concentrations [10-13]. The fact that both central and peripheral β-endorphin levels appear to change under hyperglycemic or hypoglycemic conditions suggests that endorphins are implicated in the regulation of glucose homeostasis [4,13]. Specifically, β-endorphin infusion attenuated glucose decline during prolonged exercise [6,7,9,14,15], a result that was accompanied by marked changes in glucoregulatory hormones such as insulin and glucagon whereas opiate blockade produced opposite results [6,14,15]. Thus, there is enough data to support that β-endorphin could be affected by differences in blood glucose availability as the ones produced by the consumption of different glycemic index (GI) foods.

Glycemic index ranks foods according to their effect on blood glucose levels compared to a reference food [16]. There are several studies that examined the effects of foods of various GI values prior to exercise with inconsistent results being reported in regards to performance [17-20] and carbohydrate utilization during exercise [17,19]. Exercise performance has been positively affected by low glycemic index (LGI) food [17] and remained unaffected by high glycemic index (HGI) food [18,19]. Even though there is inconsistency regarding the benefits of the ingestion of foods of varying GI on exercise performance, several findings indicate that ingestion of LGI foods may be more suitable over HGI consumption prior to prolonged exercise because they enhance carbohydrate availability during exercise [21,22].

However, the mechanisms responsible for the enhanced carbohydrate availability during exercise following LGI food ingestion are still speculative in nature. Several hormonal changes take place that modulate nutrient availability to the working muscle during exercise. Clearly, insulin, catecholamines and glucagon are the most important hormones that influence the breakdown and supply of nutrients to the muscle [23]. A decrease in insulin and an increase in catecholamines result in a higher lipolytic rate and oxidation of lipids avoiding episodes of hypoglycemia. Elevation of β-endorphin levels resulted in attenuation of blood glucose decline during prolonged exercise [9] which could be partly attributed to a higher gluconeogenic rate [8].

Therefore, the aim of this study was to examine the effects of the consumption of foods of various GI values on performance, β-endorphin levels and nutrient utilization during prolonged exercise.

## Methods

### Subjects

Eight untrained healthy males volunteers (age: 22.8 ± 3.6 yrs; height: 174.1 ± 4.2 cm; body mass: 75.1 ± 5.2 kg; body fat: 10.6 ± 3.4%; VO_2max_: 45.9 ± 6.4 ml·Kg^-1^min^-1^) participated in this study. Inclusion criteria were absence of clinical signs or symptoms of chronic disease as determined by physical examination and laboratory analyses and absence of prescribed medication. All subjects were informed about the nature of the study, the associated risks and benefits and they signed an informed consent form. Procedures were in accordance with the Helsinki declaration of 1975 and the Institutional Review Board approved the study.

### Experimental design

*VO_2max _assessment*. Each subject performed an incremental cycling test on a cycle ergometer (Monark, Vansbro, Sweden) to determine VO_2max_. The incremental cycling test to exhaustion and the accompanying gas-collection procedures have been described in detail previously [24]. Briefly, each subject started pedalling at 60 revolutions per minute (rpm) with no additional workload for 150 s. Work rate was then added incrementally every 60 s with the intent of reaching the subject's maximal exercise capacity within 6 to 12 min. VO_2max _was determined when three of the following four criteria were met: (i) volitional fatigue or inability to maintain 60 rpm, (ii) a < 2 mL^.^kg^-1.^min^-1 ^increase in VO_2 _with an increase in work rate, (iii) a respiratory exchange ratio ≥ 1.10, and (iv) a HR within 10 bpm of the theoretical maximum HR (220 - age).

The results of the initial maximal test were used to determine the exercise intensity that corresponded to 65% of each subject's VO_2max_. Gas analyzer was calibrated immediately before each subject's test. Peak oxygen consumption (VO_2_) was determined as the highest 20-s average value of VO_2 _observed over the last 60 s of exercise.

*Food consumption and exercise trial*. Each subject undertook three trials in a randomized counterbalance order with each trial separated by a period of 7 days. Subjects were asked to refrain from strenuous physical activities and maintain their customary dietary intake for 72 h prior to the testing days. To minimize the variation in glycogen levels the subjects maintained a constant diet (6-8 g. kg^-1 ^body weight of carbohydrate intake) and training schedule [25]. On the days of the main trials, subjects arrived at the laboratory at 08:00 AM, after a 10-h overnight fast. Upon arrival each subject rested quietly for at least 10 min and then an indwelling catheter was inserted in a forearm vein for blood sampling. On each occasion, after collection of the baseline data, one of the following test meals was consumed 30 min before exercise: a) 1.5 g of carbohydrates. kg^-1 ^body mass from an HGI food (white bread with strawberry jam having a glycemic index = 70), b) 1.5 g of carbohydrates. kg^-1 ^body mass from an LGI food (dried apricots having a glycemic index = 30), c) 300 ml of water alone (control). In order to preclude differences in hydration status prior to submaximal exercise participants ingested 300 ml of water prior to exercise in the two GI trials also.

Subjects had 5 min to eat the meal and rested for the next 30 min before they commenced cycling. The duration of submaximal exercise was 1 h at 65% VO_2max_. After the 1-h of cycling, the resistance increased to 90% VO_2max_, and the subjects exercised until they could no longer maintain the designated cadence (60 rpm). We assumed that 1-h of exercise at submaximal exercise intensity after the ingestion of different glycemic index foods will result in different muscle glycogen levels. This in turn could have an effect on performance when a subsequent short and intense period of exercise would follow. Therefore, the reason for increasing the intensity to 90% of VO_2max _was to exhaust subjects in a fast way. This model of assessing performance has been used in previous work that was concluded in our lab [26]. Exercise time to exhaustion (from the increase of the resistance to inability to maintain the cadence) was recorded to the nearest second. Time to exhaustion at 90% VO_2max _was reproducible in preliminary trials [coefficient of variation (CV) 6.2 ± 0.7%].

During exercise, one-minute expired air samples were collected every 10 min, and each subject drank at least 250 ml of water per 30 min to ensure adequate hydration status [27]. From VCO_2 _and VO_2 _(L^.^min^-1^) total carbohydrate and fat oxidation rates (g^.^min^-1^) were calculated for the 1-h submaximal exercise bout using published stoichiometric equations [28]. Heart rate was monitored continuously during exercise by short-range telemetry (Sports Tester PE 3000, Polar Electro, Kempele, Finland). During all trials, subjective ratings of perceived exertion (RPE) were obtained every 10 min by using the modified Borg scale [29]. All trials were conducted under conditions of similar temperature (23 ± 1°C) and relative humidity (50-60%).

### Blood collection and biochemical assays

All blood samples were drawn from a three-way valve inserted into the end of a catheter. Blood samples (10 ml) were drawn from a forearm vein before the meal, at 15 and 30 min after the meal, every 20 min during exercise (20, 40 and 60 min) and at exhaustion. Blood was allowed to clot at room temperature for 20 min and centrifuged at 1500 × *g *for 10 min. The serum layer was removed and frozen at -70°C in multiple aliquots for later analysis. All variables were analyzed in duplicates. Plasma glucose concentration was determined spectrophotometrically (Hitachi UV 2001) with commercially available kits (Spinreact, Santa Coloma, Spain). β-Endorphin and insulin were assayed by radioimmunoassay method. Blood lactate concentration was determined spectrophotometrically (Dr Lange LP 20, Berlin, Germany). Haematocrit was measured by microcentrifugation and haemoglobin was measured using a kit from Spinreact (Santa Coloma, Spain). Post exercise plasma volume changes were computed on the basis of haematocrit and haemoglobin as previously described [30]. CV for glucose, insulin, β-endorphin and lactate were 5.3%, 4.9%, 4.8% and 2.1%, respectively.

### Dietary analysis

To control for the effect of previous diet on the outcome measures of the study and establish that participants had similar levels of macronutrient intake under the three conditions, they were asked to record their diet for three days preceding each trial and repeat this diet before the second and third exercise condition. Each subject had been provided with a written set of guidelines for monitoring dietary consumption and a record sheet for recording food intake. Diet records were analyzed using the nutritional analysis system Science Fit Diet 200A (Sciencefit, Greece) and the results of the analysis are presented in Table 1.

**Table 1:** 3-day dietary analysis recall (mean ± SD)

	Control	LGI	HGI
Energy (kcal)	3559 ± 177	3627 ± 153	3721 ± 393
Carbohydrates (% energy)	51.1 ± 1.3	51.8 ± 1.1	52.4 ± 1.3
Fat (% energy)	33.3 ± 1.4	32.1 ± 1.1	31.6 ± 2.0
Protein (% energy)	15.6 ± 1.0	16.1 ± 1.6	16.0 ± 1.1

No significant differences were detected in any variable between control group, low glycemic index (LGI) group and high glycemic index (HGI) group.

#### Statistical analyses

The distribution of all dependent variables was examined by Shapiro-Wilk test and was found not to differ significantly from normal. Data are presented as mean ± SEM. Two-way ANOVA (trial × time) with repeated measurements on both factors were used to analyze the assessed parameters. If a significant interaction was obtained, pairwise comparisons were performed through simple contrasts and simple main effects analysis. One way ANOVA was used to analyze time to exhaustion, carbohydrate and fat oxidation rates.

## Results

### Exercise performance

**Figure 1:**
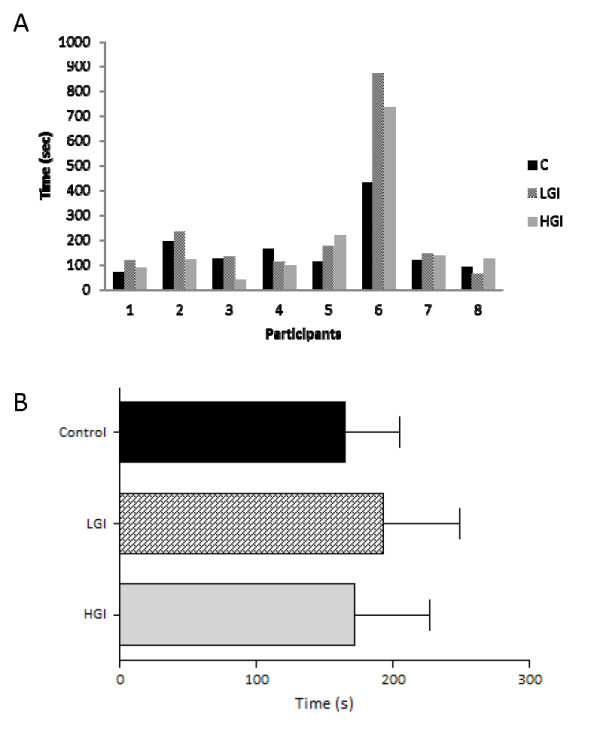
**Time to exhaustion (individual responses, A and mean values, B) after the ingestion of LGI, HGI and control meals (mean ± SEM)**. LGI: Low Glycemic Index; HGI: High Glycemic Index.

The average exercise intensity during the 1-h submaximal cycling for the control, LGI, and HGI trials were 64.9 ± 2.4%, 64.7 ± 1.9% and 65.0 ± 2.1% of VO_2max_, respectively and was not different between trials. Individual responses and mean values of time to exhaustion of the three trials after the 1-h cycling are presented in Figure 1A and 1B, respectively. Mean values of time to exhaustion did not differ between the three trials.

### RPE, heart rate and ventilation

**Figure 2:**
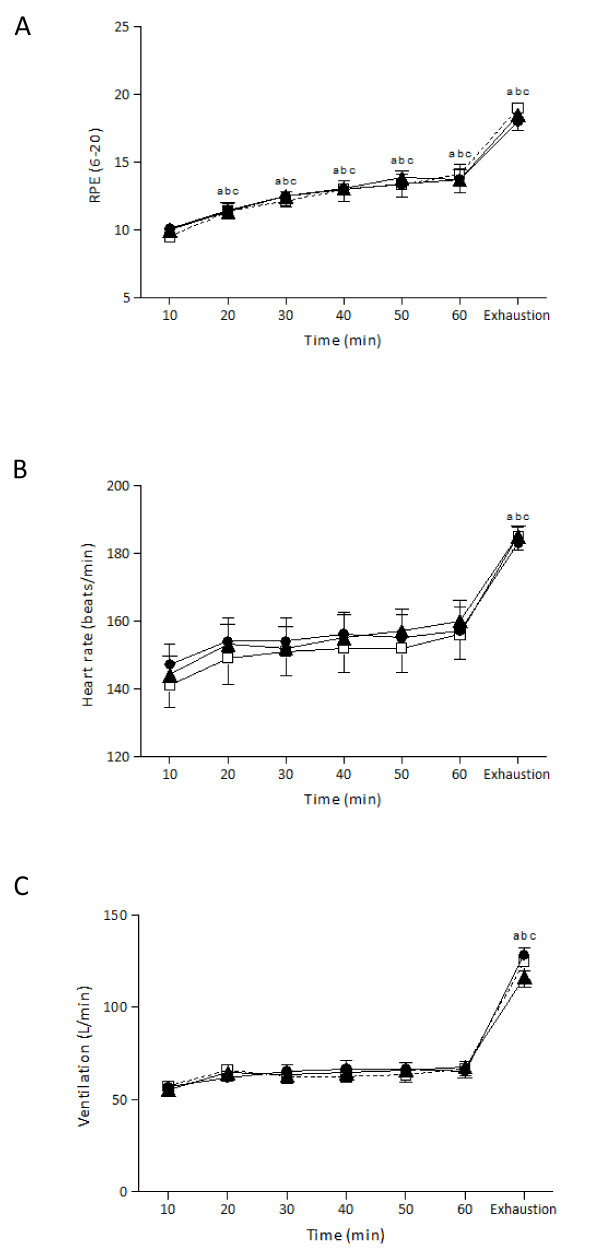
**RPE, heart rate and ventilation responses during exercise after the ingestion of LGI, HGI and control meal (mean ± SEM)**. LGI: Low Glycemic Index; HGI: High Glycemic Index.^a ^Significantly different from 10 for the HGI group (*P *< 0.05),^b ^Significantly different from 10 for the LGI group (*P *< 0.05),^c ^Significantly different from 10 for the control group (*P *< 0.05).

There was no significant main effect of trial or time by trial interaction for RPE (Figure 2A). However, there was a significant main effect of time (*P *< 0.001, *η^2 ^*= .98, observed power = 1.00). RPE levels increased significantly at 20 min and remained significantly elevated until exhaustion for all trials. There were no significant differences at rest between the three trials for heart rate (Control = 68.0 ± 2.6 bpm, LGI = 66.3 ± 4.2 bpm, HGI = 66.5 ± 3.4 bpm). There was no significant main effect of trial or time by trial interaction for heart rate (Figure 2B) and ventilation (Figure 2C). However, there was a significant main effect of time for heart rate (*P *< 0.001, *η^2 ^*= .97, observed power = 1.00), and ventilation (*P *< 0.001, *η^2 ^*= .98, observed power = 1.00). Pairwise comparisons revealed significant differences between the 10 min and exhaustion time points for all trials for heart rate and ventilation.

### Substrate oxidation

**Figure 3:**
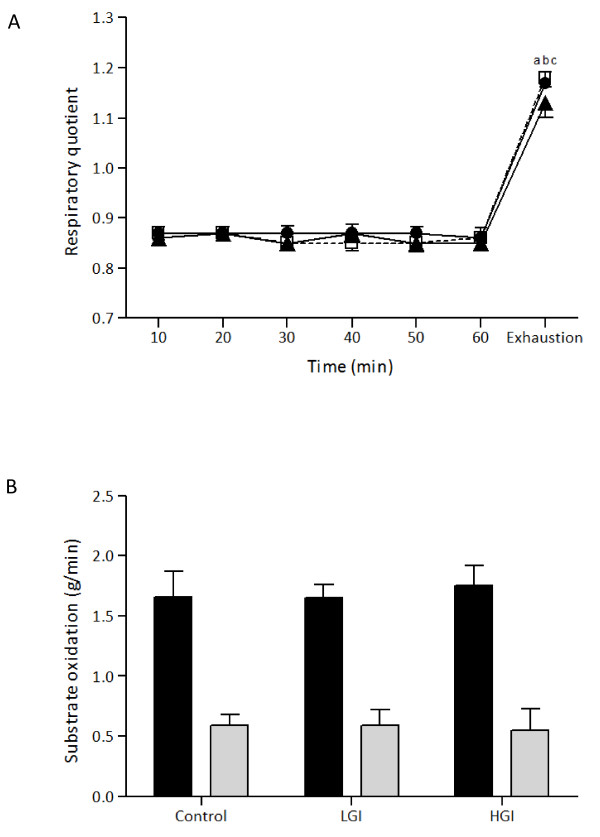
**Respiratory quotient and substrate oxidation rate during exercise after the ingestion of LGI, HGI and control meal (mean ± SEM)**. LGI: Low Glycemic Index; HGI: High Glycemic Index.^a ^Significantly different from 10 for the HGI group (*P *< 0.05),^b ^Significantly different from 10 for the LGI group (*P *< 0.05),^c ^Significantly different from 10 for the control group (*P *< 0.05).

There was no significant main effect of trial or time by trial interaction for respiratory quotient (RQ; Figure 3A). However, there was a significant main effect of time (*P *< 0.001, *η^2 ^*= .97, observed power = 1.00). RQ appeared significantly elevated only at exhaustion with no significant difference between the three trials. Carbohydrate and fat oxidation rates (Figure 3B) was not different between the three trials during exercise.

### Lactate, glucose and insulin

**Figure 4:**
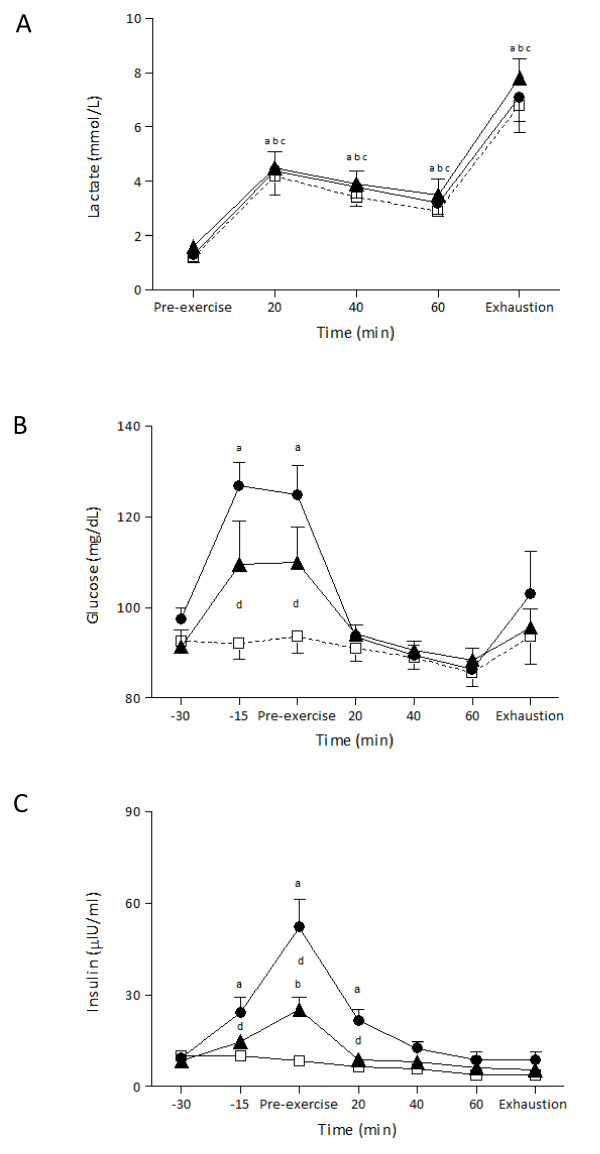
**Lactate, glucose and insulin responses during exercise after the ingestion of LGI, HGI and control meal (mean ± SEM)**. LGI: Low Glycemic Index; HGI: High Glycemic Index.^a ^Significantly different from the first time point for the HGI group (*P *< 0.05),^b ^Significantly different from the first time point for the LGI group (*P *< 0.05),^c ^Significantly different from the first time point for the control group (*P *< 0.05);^d ^significantly different between HGI and control group at the same time point (*P *< 0.05).

There was no significant main effect of trial or time by trial interaction for lactate (Figure 4A). However, there was a significant main effect of time (*P *< 0.001, *η^2 ^*= .92, observed power = 1.00). Lactate levels increased significantly at 20 min of exercise and remained significantly elevated until exhaustion for all trials.

Plasma glucose levels (Figure 4B) showed significant differences for time (*P *< 0.001, *η^2 ^*= .75, observed power = 1.00) and for trial by time interaction (*P *< 0.01, *η^2 ^*= .60, observed power = .90). Plasma glucose levels were significantly higher in HGI at 15 and 30 min after the ingestion of the meal compared with those of LGI and control. After 20 min of exercise plasma glucose levels fell to pre-exercise levels and remained unchanged until the end of exercise. No significant differences were noted between the control and LGI trials in glucose levels.

Plasma insulin levels (Figure 4C) showed significant differences for time (*P *< 0.001, *η^2 ^*= .85, observed power = 1.00) and for trial by time interaction (*P *< 0.001, *η^2 ^*= .79, observed power = 1.00). Plasma insulin levels increased significantly above baseline values 15 and 30 min after the HGI and LGI meals. However, the rise was smaller after the LGI meal compared with the rise after the HGI meal. That increase was significantly different compared to the plasma insulin levels of control trial at the respective time points. By 20 min of exercise insulin levels had significantly decreased in both HGI and LGI trials. However, at this time point plasma insulin levels were significantly higher in HGI compared to control trial. No significant differences were noted between the three trials at 60 min and at exhaustion.

### β-Endorphin

**Figure 5:**
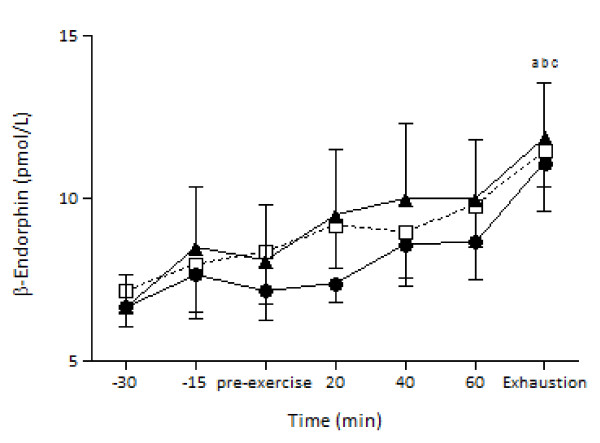
**β-Endorphin responses during exercise after the ingestion of LGI, HGI and control meal (mean ± SEM)**. LGI: Low Glycemic Index; HGI: High Glycemic Index.^a ^Significantly different from -30 for the HGI group (*P *< 0.05),^b ^Significantly different from -30 for the LGI group (*P *< 0.05),^c ^Significantly different from -30 for the control group (*P *< 0.05).

There was no significant main effect of trial or time by trial interaction for β-endorphin (Figure 5). However, there was a significant main effect of time (*P *< 0.05, *η^2 ^*= .86, observed power = 1.00). β-Endorphin increased significantly at the end of the exercise and that response was evidenced in all three trials.

## Discussion

The present study indicates that ingestion of foods of different GI values 30 min prior to exhaustive cycling exercise does not result in significant changes in exercise performance. Furthermore, consumption of carbohydrates of LGI and HGI does not alter β-endorphin levels during exercise and does not result in significant changes in carbohydrate and fat oxidation rate during exercise.

Ingestion of carbohydrates prior to exercise resulted in an increase in glucose and insulin (Figure 4A and 4B). It is well known that when blood glucose increases the pancreatic beta cells increase their output of insulin in order to facilitate glucose uptake by the tissues. In our study an initial increase of glucose was observed and then plateaued whereas insulin continued to increase up to 30 minutes following the ingestion of foods. The same glucose and insulin response prior to exercise was seen in De Marco et al. study when the same amount of carbohydrates was ingested [17]. This response of glucose and insulin is common since the initial increase in glucose constitutes the main stimulus for the delayed insulin increase.

Several studies attempted to alter the carbohydrate composition of a meal prior to exercise in an effort to improve performance. A number of those studies show no improvement in exercise performance [19,22,31-33]. Febbraio et al. [19] utilized a similar design with the one employed in this study and found no significant differences in exercise performance. Subjects received low and high glycemic foods (1.0 g. kg^-1 ^of body weight) 30 min prior to a 120-min submaximal exercise bout that was followed by a 30 min time trial. Total work performed during the time trial was similar between the LGI, the HGI and the control condition. These results were evident despite the fact that carbohydrate oxidation was greater during the HGI condition. No significant differences in exercise performance were noted in two other studies by the same group [31,32] when subjects received LGI and HGI foods (1.0 g. kg^-1 ^of body mass) 45 min prior to submaximal exercise that was followed again by a time trial. Although differences in glucose and insulin levels were reported following consumption of the LGI and HGI prior to exercise, there were no apparent differences in the blood metabolites during the steady state exercise. Thomas et al. [33] used four meals with different glycemic index foods (30, 36, 73 and 100) that each provided 1.0 g. kg^-1 ^of body weight. The meal was consumed 1 h prior to cycling to exhaustion at 65-70% of VO_2max_. The results showed no significant differences in time to exhaustion between trials. No enhancement in exercise performance was found when low and high glycemic index foods were provided 3 h prior to exercise even though there was a relative shift in substrate utilization from carbohydrate to fat following the LGI meal [22]. As far as exercise performance is concerned, results from the present study coincide with those of earlier reports suggesting that although pre-exercise GI manipulation affects pre-exercise glucose and insulin levels, it does not presumably influence the rate of muscle glycogen utilization or exercise performance. Differences in glucose levels and carbohydrate and fat oxidation during steady state exercise could influence exercise performance during a subsequent short and intense exercise. Evidence indicates that increasing fat oxidation leads to sparing of glycogen [34] and spared glycogen or higher blood glucose levels towards the end of exercise could be used to allow for high-intensity exercise to be continued for a longer time affecting exercise performance. In a recent study where low and high GI foods were consumed 15 minutes prior to exercise LGI food resulted in higher glucose levels at the end of exercise and performance was greater compared to a HGI food and a placebo condition [35]. However, it has to be noted that the subjects in this study were not professional athletes and an abrupt increase in the exercise intensity following a steady state exercise could not be able to reveal performance and metabolic responses accurately. This is a limitation of the present study and further research should explore performance, metabolic and β-endorphin responses in well-trained athletes with a different time trial design (i.e. continues exercise at a submaximal intensity).

On the other hand, there are several studies that examined the effects of different GI foods, at different times prior to exercise, on exercise performance and substrate metabolism that suggest an improvement of exercise performance following LGI food consumption prior to exercise [17,36-40]. Thomas et al. [36] were amongst the first ones that expressed interest in the role of GI in sports nutrition. In their study, participants under four different conditions received three foods of different GI and water. Each meal provided 1.0 g. kg^-1 ^of body weight and was given 60 min prior to cycling to exhaustion at 65-67% VO_2max_. A significant 20 min prolonged workout was performed after consumption of the LGI foods that was accompanied by more stable glucose levels and higher free fatty acid concentration during exercise. De Marco et al. [17] also showed a 59% increase in time to exhaustion after a 2-h submaximal bout in a LGI trial compared with a HGI trial accompanied by a relative hyperglycemia and lower RPE and RQ [17]. Moore et al. [38] administered low and high GI foods 45 min prior to a 40 km cycling trial and found a significantly improved performance following the LGI trial. Higher glucose levels at the end with no differences in carbohydrate and fat oxidation rates were noted between the two trials. In the study of Little et al. [37], improved performance also appeared following the consumption of LGI and HGI foods (1.3 g. kg^-1 ^of body weight) after the end of a simulated soccer game [37]. Finally, consumption of HGI food (1.0 g. kg^-1 ^of body weight) resulted in a 12.8% increase in time to exhaustion compared to a placebo trial [20]. Discrepancies seen in the results reported by the aforementioned studies may be attributed to differences in meals' time of ingestion, amounts of foods (per kilogram of body weight) or methods of assessment of exercise performance.

In order to provide the same hydration status prior to each exercise trial subjects ingested the same amount of water (300 ml). However, the subjects during the GI trials ingested more volume (300 ml + GI meal) as compared to the control trial (300 ml). Eventhough the different ingested volume could affect gastric emptying and subsequently the metabolic responses this seems unlikely since none of the metabolic variables assessed in the control trial were changed prior to exercise. However, the different ingested volume between the control and the GI trials could have an effect during exercise and this is something that needs further attention in future investigations.

Previous research indicates a role of β-endorphin in metabolism and fatigue perception during exercise. For example, Fatouros et al. [4] manipulated the carbohydrate intake of rats and found a higher concentration of β-endorphin in plasma and hypothalamus indicating that this peptide is affected by nutritional factors at peripheral and central level. Furthermore, manipulating the levels of peripheral β-endorphin by infusion of this opioid resulted in significant changes in glucose levels and pancreatic hormones during exercise further indicating that β-endorphin has effects on carbohydrate metabolism [6,7,9]. Therefore, it was worth examining whether intake of carbohydrates of different quality (as far as glucose response is concerned) will result in different responses in β-endorphin at rest and/or during exercise. The results from the present study indicate that ingestion of different GI foods does not result in different β-endorphin levels at rest and during exercise. β-endorphin is rapidly responding to an intense bout of exercise [41]. It was hypothesized that differences in GI foods would affect metabolism leading to different glycogen levels allowing for higher work output. More intense work, in turn, could lead to different beta endorphin responses. This hypothesis was rejected since no differences in performance or beta endorphin levels were observed.

One reason for the inability to observe significant differences in β-endorphin at rest following the consumption of GI foods could be related to the amount of carbohydrate consumed. Subjects received carbohydrates equivalent to 1.5 g. kg^-1 ^of body weight and it seems that this amount of carbohydrates is not enough to alter the response of the pituitary and hypothalamus in the release of β-endorphin. Only one other study examined the response of β-endorphin to carbohydrate and fat meals and found similar results with this study since β-endorphin response changed in the obese but not in individuals of normal weight [5]. β-Endorphin did not increase significantly until at the exhaustion time point. The inability of β-endorphin to increase during submaximal exercise could be related to the exercise intensity [10]. Previous research indicates that β-endorphin contributes to the modulation of pain perception and fatigue during exercise [42]. The results from this study revealed no differences in RPE and β-endorphin levels between the three trials contradicting the results from the aforementioned study.

## Conclusion

In conclusion, ingestion of different GI foods of the same quantity did not result in differences in exercise performance or β-endorphin responses at rest and during exercise. Future studies should look into the effects of altering the amount of ingested GI foods and the time of ingestion on β-endorphin responses at rest and during exercise. Finally, increasing the number of participants and testing trained subjects or athletes are additional factors that should be taken into consideration prior to designing similar studies.

## Competing interests

The authors declare that they have no competing interests.

## Authors' contributions

AZJ conceived of the study, collected and analysed data, and wrote the manuscript. TT collected and analysed data. IF participated in the design of the study, analysed data and reviewed the manuscript. MGN analysed data and performed the statistical analysis. VP analysed data and reviewed the manuscript. CY collected and analysed data. SR analysed data. YK reviewed the manuscript. All authors reviewed and approved the manuscript.
